# Supplementary material for: Content-rich biological network constructed by mining PubMed abstracts
Source: BMC Bioinformatics. 2004 Oct 8;5:147. doi: 10.1186/1471-2105-5-147 (PMC528731; doi:10.1186/1471-2105-5-147)
Supplement: Additional File 5 — The original Chilibot query results of the term "long-term potentiation (LTP)" and 22 other terms, limiting the latest references analyzed to the years 1990, 1995, 2000, and 2004. [file 1471-2105-5-147-S5.bz2 › chilibotAdditionalFile5/ltp2000/html/TRKA_ERK.html]

 


 **TRKA** and **ERK** 
  
Found 22 abstracts in PubMed,  **22 abstracts were retrieved and analyzed**.  


---

 Search Google  |
 PDF files only 
|  EDU domain only 

---

**Interactive relationship** (e.g. stimulation, inhibition, etc)

- NGF stimulation of  **erk**  phosphorylation is impaired by a point mutation in the transmembrane domain of  **trkA**  receptor.  Ref: 10854038 J Mol NeurosciJ Mol Neurosci, 2000
- Furthermore, the unglycosylated active  **TrkA**  receptors are unable to activate kinases in the Ras MAP kinase pathway, MEK and  **Erk** .  Ref: 10235685 J Neurobiol, 1999
- NGF induced phosphorylation of the  **trkA**  receptor, activated a cascade of cellular intermediaries such as phospholipase C gamma 1 and  **ERK**  proteins, and stimulated c fos gene transcription in all  **trkA**  expressing clones.  Ref: 7761093 Oncogene, 1995
- In addition, we show that high K treatment did not induce Trk A  [ **TRKA** ]  phosphorylation, thus excluding the possibility of calcium operating through this receptor to activate the  **ERK**  signal transduction pathway.  Ref: 9603222 J Neurochem, 1998
- These data suggest that calmodulin mediates  **ERK**  activation induced by increases in intracellular calcium concentration in PC12 cells by a mechanism that seems to be independent of Trk A  [ **TRKA** ]  and EGFR activation.  Ref: 9603222 J Neurochem, 1998

- :-)

  **Parallel relationship** (e.g. studied together, co-existance, homology, etc.)

  - The results of this study suggest that PI3 K may be required for the signals initiated by  **TrkA**  internalization and demonstrate that specific endocytic events may distinguish  **ERK**  signaling via Rap1 and Ras.  Ref: 11027277 Mol Cell Biol, 2000
  - The data also indicate that NGF increased phosphorylation of  **trkA**  and the mitogen activated protein kinase extracellular signal regulated kinase  **ERK**  in dentate gyrus in vitro.  Ref: 10560922 Hippocampus, 1999
  - Activation of signal transduction pathways involving  **trkA** , PLCgamma 1, PKC isoforms and  **ERK**  1 2 by tetanus toxin.  Ref: 10996319 FEBS Lett, 2000
  - We found that MED283  **TrkA**  cells expressing a dominant negative Ras inhibitor N17Ras failed to undergo  **ERK**  activation and apoptosis following NGF treatment.  Ref: 10617652 J Biol Chem, 2000
  - Western blot analysis of these cells revealed tyrosine phosphorylation of the high affinity NGF receptor  **TrkA**  gp140 and activation of a downstream signal cascade element,  **ERK**  1 MAP kinase.  Ref: 9630563 Brain Res Mol Brain Res, 1998
  - In the present work the differential activation of PKC isoforms and of signal transduction pathways, including nerve growth factor receptor  **trkA** , phospholipase Cgamma 1 PLCgamma 1, and extracellular regulated kinases 1 and 2  **ERK**  1 2 by TeTx in a synaptosome enriched P 2 fraction from rat brain is reported.  Ref: 10996319 FEBS Lett, 2000
  - NGF stimulation of HMC 1 cells induced tyrosine phosphorylation of  **TrkA**  protein, increased expression of the early response genes c fos and NGF1 A, and activation of  **ERK**  mitogen activated protein kinase, results which indicate that  **TrkA**  receptors in HMC 1 cells are fully functional.  Ref: 9292513 Blood, 1997
  - Analysis of phosphorylation of the two tyrosine kinase substrates  **trkA**  and  **ERK**  in synaptosomes prepared from untetanized and tetanized dentate gyrus revealed that LTP was associated with increased phosphorylation of both proteins.  Ref: 10560922 Hippocampus, 1999
  - Like suramin and NGF, PPS induced neurite outgrowth from the PC12 cell line which correlated with tyrosine phosphorylation of the high affinity NGF receptor  **TrkA**  gpl40 and  **ERK**  1 MAP kinase.  Ref: 9874182 J Neurooncol, 1998
